# First-trimester fasting plasma glucose as a predictor of gestational diabetes mellitus and the association with adverse pregnancy outcomes

**DOI:** 10.12669/pjms.35.1.216

**Published:** 2019

**Authors:** Ping Li, Shuo Lin, Ling Li, Jinhui Cui, Shuisheng Zhou, Jianhui Fan

**Affiliations:** 1 Ping Li, MD. Department of Obstetrics and Gynecology, The Third Affiliated Hospital of Sun Yat-Sen University, Guangzhou 510630, China; 2 Shuo Lin, DD. Department of Endocrinology, The Third Affiliated Hospital of Sun Yat-Sen University, Guangzhou 510630, China; 3 Ling Li, MD. Department of Obstetrics and Gynecology, The Third Affiliated Hospital of Sun Yat-Sen University, Guangzhou 510630, China; 4 Jinhui Cui, MD. Department of Obstetrics and Gynecology, The Third Affiliated Hospital of Sun Yat-Sen University, Guangzhou 510630, China; 5 Shuisheng Zhou, MD. Department of Obstetrics and Gynecology, The Third Affiliated Hospital of Sun Yat-Sen University, Guangzhou 510630, China; 6 Jianhui Fan, MD. Department of Obstetrics and Gynecology, The Third Affiliated Hospital of Sun Yat-Sen University, Guangzhou 510630, China

**Keywords:** Gestational diabetes mellitus, Fasting plasma glucose, Predicting, Adverse pregnancy outcomes

## Abstract

**Objective:**

To evaluate the usefulness of a fasting plasma glucose (FPG) at the first trimester in predicting gestational diabetes mellitus (GDM) and the association between FPG and adverse pregnancy outcomes.

**Methods:**

The levels of FPG in women with singleton pregnancies were measured at 9-13^+6^ weeks. A two hour 75-g oral glucose tolerance test (OGTT) was completed at 24-28 weeks and the International Association of Diabetes and Pregnancy Study Groups (IADPSG) criteria was used. Adverse pregnancy outcomes were assessed and recorded.

**Results:**

Among 2112 pregnant women enrolled in the study, 224 (10.6%) subjects were diagnosed with GDM. The AUC for FPG in predicting GDM was 0.63 (95% CI 0.61- 0.65) and the optimal cutoff value was 4.5 mmol/L (sensitivity 64.29% and specificity 56.45%). Higher first-trimester FPG increased the prevalence of GDM, large for gestational age (LGA) and assisted vaginal delivery and/or cesarean section (all *P* < 0.05).

**Conclusion:**

FPG at first trimester could be used to predict GDM and higher first-trimester FPG was associated with adverse pregnancy outcomes.

## INTRODUCTION

Gestational diabetes mellitus (GDM) is one of the most common complications of pregnancy and the incidence of GDM is increasing globally.^1^,^2^ Women with GDM are associated with many maternal (preeclampsia, cesarean section, birth injuries) and fetal consequences (macrosomia, hypoglycemia, shoulder dystocia).^3^,^4^ Commonly, GDM can be diagnosed by using the oral glucose tolerance test (OGTT) during 24-28 weeks of gestation. However, maternal metabolic status at the early stage of pregnancy may affect maternal and perinatal outcomes.^5^ appropriate diet and medication interventions can reduce the incidence of GDM.^6^,^7^ Therefore, early detection of women at high risk of GDM is clinically important.

Most researches focused on identifying risk factors at the first trimester for GDM development, including family predisposition, increased maternal age, cultural background, high Body Mass Index (BMI), elevated C-reactive protein levels and history of fetal macrosomia.^8^ Fasting plasma glucose (FPG) is a predictive index for type 2 diabetes. It is easy to administer, well tolerated, inexpensive and reproducible. GDM is like type 2 diabetes in many aspects. The efficiency of FPG in predicting GDM is no universally agreed as different criteria are applied for the diagnosis and various gestational weeks or races are chosen. Previous studies had showed that FPG could be used to predict risk for GDM in later pregnancy.^9^,^10^ In our study, we tried to determine the accuracy of first-trimester FPG in predicting GDM using the International Association of Diabetes and Pregnancy Study Groups (IADPSG) criteria^11^ and find out whether FPG at early trimester was associated with the maternal and neonatal adverse outcomes.

## METHODS

This retrospective study was approved by the Human Research Ethics Committee of the third affiliated hospital of Sun Yat-Sen University. Medical records of 2112 singleton pregnant women were collected from the third affiliated hospital of Sun Yat-Sen University, Guangzhou, China, from January 2016 to June 2017. Women with already diagnosed pregestational diabetes were excluded. All women had the first prenatal visit during 9-13^+6^ gestation weeks and then received regular prenatal services and delivered in this hospital.

All patients were underwent FPG test during 9-13^+6^ gestation weeks after at least 8 hours fasting and glucose oxidase method were used to assay. A two hour 75-g OGTT was performed between 24-28 weeks and the diagnosis criteria was based on the IADPSG^12^ (i.e., one or more plasma venous glucose values ≥ 0 h, 5.1 mmol/L; 1 h, 10.0 mmol/L; or 2 h, 8.5 mmol/L).

We recorded patients’ baseline characteristics when FPG test was done, including age, parity and pregestational body mass index (BMI) (BMI=weight (kg) / height^2^ (m^2^)). After delivery, obstetric and neonatal data were collected, including gestational age at delivery, delivery mode, birth weight and one and five minute Apgar score of neonate. Adverse pregnancy outcomes were assessed and recorded, including preterm delivery, premature rupture of membranes (PROM), pregnancy induced hypertension (PIH), intrauterine growth restriction (IUGR), polyhydramnios, postpartum hemorrhage (PPH), macrosomia, large for gestational age (LGA) and low Apgar score.

Preterm delivery was defined as a birth before 37 weeks gestation. IUGR was defined as a fetal weight less than 10th percentile for gestational age. PPH was defined as postpartum hemorrhage more than 500ml for natural birth or more than 1000ml for cesarean section. Polyhydramnios was defined as amniotic fluid index (measure of four quadrants) higher than 95th percentile for gestational age. Macrosomia was defined as a birthweight higher than 4.0 kg. LGA was defined as a birthweight larger than the 90th percentile for gestational age by gender. Low Apgar score was defined as Apgar score less than 7 at one or five minutes.

### Statistical Analysis

SPSS-19.0 (SPSS, Inc., Chicago, IL) was used for analysis. Continuous variables were presented as mean (SD), skewed variables as medians (interquartile range) and categorical variables as proportions. Difference in variables between groups was analyzed using t test, Mann-Whitney test or Chi-square test. The area (AUC) under the receiver operating characteristic (ROC) curve was used to evaluate the performance of FPG to predict GDM and the optimal cut-off point was calculated. DeLong test^13^ was used to compare areas under ROC curves. The sensitivity, specificity, positive (PPV) and negative (NPV) predictive values for different threshold values of FPG along with likelihood ratios of positive (LR+) and negative (LR-) tests were calculated. Multivariate logistic regression analysis was utilized to explore the independent associated factors of GDM (backward method was used). *P* < 0.05 was considered statistically significant.

### Ethical Approval

All procedures performed in studies involving human participants were in accordance with the ethical standards of the institutional and/or national research committee and with the 1964 Helsinki declaration and its later amendments or comparable ethical standards.

## RESULTS

A total of 2112 women were included in this study. Of them, 224 (10.6%) subjects were diagnosed with GDM. The characteristics of participants were shown in Table-I. Compared with normal group, subjects in GDM group were older and more multiparous (*P* < 0.001). They also delivered earlier (39.00 vs. 39.29, *P*=0.001), needed more assisted vaginal delivery or cesarean section (43.8% vs. 35.0%, *P*=0.010) and had more postpartum hemorrhage volume (*P*=0.001). The first-trimester FPG was higher (*P* < 0.001) but maternal BMI gain was lower (*P*=0.003) in GDM group. Low Apgar score (≤7 at 1 or 5 minutes) was also more prevalent in GDM group than that in normal group (3.1% vs 1.2%, *P*=0.022).

**Table-I:** Characteristics of the study participants*.

Characteristics	Total (n=2112)	Normal (n=1888)†	GDM (n=224)	P
Maternal age (years)	30(27-34)	30(27-33)	33(29-36)	0.000
Parity	1(1-2)	1(1-2)	2(1-2)	0.000
Maternal BMI (kg/m^2^)				
Pregestation	20.03(18.75-21.59)	20.00(18.75-21.48)	20.73(2.50)	0.159
Delivery	25.78(24.07-27.56)	25.78(24.09-27.64)	25.50(2.55)	0.373
Gain	5.54(1.87)	5.77(4.58-6.86)	4.76(1.97)	0.003
FPG (mmol/L)	4.43(4.19-4.67)	4.41(4.18-4.65)	4.60(4.30-4.86)	0.000
75-g OGTT (mmol/L)				
0 hour	3.94(4.13-4.35)	4.11(3.93-4.32)	4.43(4.16-4.77)	0.000
1 hour	7.24(6.19-8.39)	7.04(6.05-8.06)	10.07(9.24-10.70)	0.000
2 hour	6.45(5.64-7.35)	6.30(5.56-7.03)	8.94(8.51-9.64)	0.000
Gestational age at delivery (weeks)	39.29(38.57-40)	39.29(38.57-40.14)	39.00(38.43-39.71)	0.001
Birth method (assisted vaginal delivery or cesarean section) (%)	35.9(758)	35(660)	43.8(98)	0.010
Postpartum hemorrhage volume (ml)	310 (255-380)	305 (255-380)	330 (265-420)	0.001
Neonatal birth weight (kg)	3.20 (2.95-3.50)	3.20(2.95-3.50)	3.20(2.95-3.55)	0.092
Low Apgar score (≤ 7 at 1 or 5 minutes) (%)	1.4(30)	1.2(23)	3.1(7)	0.022

* Values are mean (SD), medians (interquartile ranges) or percentage,

† Compared with GDM group BMI, body mass index; FPG, fasting plasma glucose; OGTT, oral glucose tolerance test.

The independent risk factors for predicting GDM by using multivariate logistic regression analysis, including the pre BMI, first-trimester FPG, maternal age and parity as confounders are shown in Table-II. First-trimester FPG and maternal age were independent risk factors and the odd ratios were 2.847 (95% CI 1.508-5.374) and 1.156 (95% CI 1.20-3.72).

**Table-II:** Risk factors for predicting GDM by using multiple logistic regression (use first-trimester FPG, preBMI, maternal age and parity as confounders).

Items	B	S.E.	Wald	P-value	Odd ratio (95% CI)
Maternal age	0.145	0.046	9.738	0.002	1.156(1.055, 1.266)
Fast plasma glucose	1.046	0.324	10.420	0.001	2.847(1.508, 5.374)

FPG, fasting plasma glucose; GDM, Gestational diabetes mellitus; preBMI, pregestational body mass index; OR, odds ratio.

**Fig.1:**
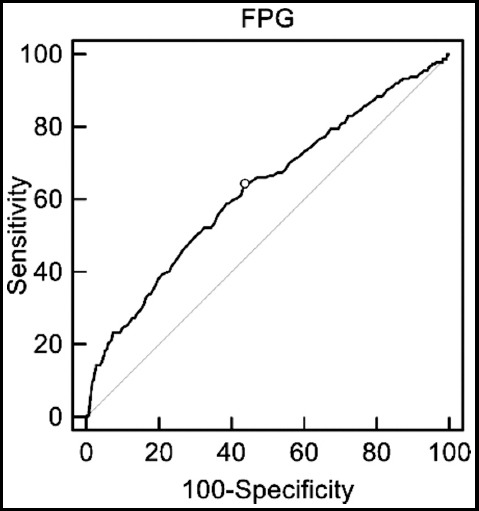
Receiver-operator characteristic curves for fasting plasma glucose in predicting gestational diabetes mellitus.

Fig.1 shows the ROC curves for determining the screening accuracy of first-trimester FPG for GDM and the AUC was 0.63 (95% CI 0.61- 0.65). Table-III selected threshold values for FPG and the associated sensitivity, specificity, PPV and NPV, and LR+ and LR–. The optimal cutoff point of FPG was 4.5 mmol/L in ROC curve which provided the highest combination of sensitivity (64.29%) and specificity (56.45%).

**Table-III:** Fasting plasma glucose at the first trimester as a predictor for gestational diabetes mellitus.

Cut point (mmol/L)	Sensitivity (%) (95% CI)	Specificity (%) (95% CI)	+LR	-LR	PPV(%)	NPV(%)
4.1	90.18(85.5 - 93.7)	17.23(15.6 - 19.0)	1.09	0.57	11.4	93.7
4.2	83.04(77.5 - 87.7)	26.80(24.8 - 28.9)	1.13	0.63	11.8	93.0
4.3	75.89(69.7 - 81.3)	36.63(34.5 - 38.8)	1.20	0.66	12.4	92.8
4.4	66.52(59.9 - 72.7)	48.68(46.4 - 51.0)	1.30	0.69	13.3	92.5
4.5 *	64.29(57.6 - 70.6)	56.45(54.2 - 58.7)	1.48	0.63	14.9	93.0
4.6	50.45(43.7 - 57.2)	69.34(67.2 - 71.4)	1.65	0.71	16.3	92.2
4.7	39.29(32.8 - 46.0)	79.23(77.3 - 81.0)	1.89	0.77	18.3	91.7
4.8	28.57(22.8 - 35.0)	86.05(84.4 - 87.6)	2.05	0.83	19.5	91.1
4.9	23.21(17.9 - 29.3)	91.28(89.9 - 92.5)	2.66	0.84	24.0	90.9
5.0	18.30(13.5 - 24.0)	94.71(93.6 - 95.7)	3.46	0.86	29.1	90.7

* Optimal cutoff point, which showed the highest combination of sensitivity and specificity +LR, positive likelihood ratio; -LR, negative likelihood ratio;

NPV, negative predictive value; PPV, positive predictive value; CI, confidence interva

The associations between first-trimester FPG and adverse pregnancy outcomes are presented in Table-IV by dividing into two groups according to the lower and upper quartiles (<4.19 or >=4.67 mmol/L) of FPG. It showed that the prevalence of GDM were significantly increased as FPG elevated at the first trimester (17.5% vs 6.8%, χ^2^ = 28.503, *P* < 0.001). The prevalences of LGA (19.0% vs. 11.8%, χ^2^ = 10.602, P = 0.001) and the assisted vaginal delivery / cesarean section (39.9% vs. 29.2%, χ^2^ = 13.510, *P*<0.001) were also increased. The prevalences of PIH, IUGR, polyhydramnios, PPH and low Apgar score were higher in the upper quartile group though there were no statistical differences between them.

**Table-IV:** The relationship between fasting plasma glucose at the first trimester and adverse pregnancy outcomes.

Obstetric and neonatal outcomes	FPG < 4.19 mmol/l (lower quartile, n=518) (%)	FPG ≥ 4.67 mmol/l (upper quartile, n=536) (%)	χ^2^	P
*Mother*				
GDM	35(6.8)	94(17.5)	28.503	0.000
PIH	6(1.2)	11(2.1)	1.327	0.249
IUGR	6(1.2)	7(1.3)	0.047	0.828
Polyhydramnios	11(2.1)	17(3.2)	1.130	0.288
PROM	143(27.6)	117(21.8)	4.732	0.030
Premature delivery (<37 weeks)	19(3.7)	19(3.5)	0.012	0.915
PPH	16(3.1)	21(3.9)	0.535	0.465
Birth method (assisted vaginal delivery or cesarean section)	151(29.2%)	214(39.9%)	13.510	0.000
*Newborn*				
Macrosomia	7(1.4)	16(3.0)	3.294	0.070
LGA	61(11.8)	102(19.0)	10.602	0.001
Low Apgar score (≤7 at 1 or 5 minutes)	4(0.8)	5(0.9)	0.080	0.777

FPG, fasting plasma glucose; GDM, Gestational diabetes mellitus; PIH, pregnancy induced hypertension; IUGR, intrauterine growth restriction; PROM, premature rupture of membranes; PPH, postpartum hemorrhage; LGA, large for gestational age.

## DISCUSSION

In the present study, we demonstrated that FPG at the first trimester could be used to predict GDM in Chinese women. According to the ROC curves, a FPG level ≥4.5 mmol/L showed an optimal combination of sensitivity (64.29%) and specificity (56.45%) for predicting GDM. Multivariate logistic regression analysis also revealed that first-trimester FPG was an independent risk factor for GDM development. In addition, higher first-trimester FPG was associated with adverse pregnancy outcomes.

The performance of first-trimester FPG as a predicting index for GDM is still controversial. The potential problems are highly dependent on the diagnostic criteria for GDM^14^,^15^ and ethnic difference. In Bhattacharya’s study, they found that FPG did not predict GDM in later pregnancy using the “two-step approach” for GDM diagnosed.^16^ In Riskin’s study,^9^ by using a 3h 100-g glucose tolerance test and the Carpenter and Coustan criteria,^17^ they concluded that higher first-trimester fasting glucose could be used as a predictor for the development of GDM among young pregnant women in Israel. In the study of Sacks,^18^ though they concluded that the specificity of FPG for screening GDM in the first trimester was poor by using a one hour 50-g glucose challenge test (GCT), the AUC was 0.7 which meant FPG still had the diagnostic accuracy for predicting GDM (AUC >0.5). In China, using a 2h 75-g OGTT and the IADPSG criteria, Min Hao et al.^19^ found FPG could be used in predicting suspicious GDM patients in the first trimester. An extensive study by Zhu et al.,^20^ involving 17186 women from China using the IADPSG criteria, showed that the first prenatal visit FPG correlated strongly with GDM at 24-28 weeks gestation. In our study, we also found similar diagnostic accuracy of FPG for predicting GDM when the IADPSG criteria was used.

Though there is no uniform worldwide optimal cut-off point for first-trimester FPG in predicting GDM, the results from different studies are still very close. Riskin-Mashiah^9^ found that the optimal threshold value of FBG was 4.6 mmol/L (with sensitivity of 65.2% and specificity of 67.6%) at the first trimester in predicting GDM. Min Hao^19^ (with a sensitivity of 53.89 and specificity of 70.90%) also found that first–trimester FPG level ≥ 4.6 mmol/L was the best threshold for predicting GDM. In this study, the optimal cut-point of FPG was 4.5 mmol/L for predicting GDM at the first trimester, with the highest combination of sensitivity and specificity. These results suggested the use of FPG for predicting GDM was reasonable and reproducible. However, different studies have reported various FPG levels may due to study population, ethnicity and diagnostic criteria. Further studies need to be conducted for the optimal threshold of FPG.

Maternal metabolic status at the early stage of pregnancy may affect maternal and perinatal outcomes.^5^ Riskin-Mashiah et al.^10^ reported that mild hyperglycemia during early pregnancy could lead to adverse outcomes. They found a strong association between first-trimester maternal fasting glycemia and the development of GDM. Large for gestational age (LGA) and/or macrosomia were also increasing with increasing fasting glycemia category. However, no significant associations were found between fasting glucose and either preterm delivery (< 37 weeks) or neonatal intensive care unit admission. Another large study found that first-trimester fasting glucose was associated with adverse pregnancy outcomes including GDM, LGA and/or macrosomic neonate and primary cesarean section.^16^ In accordance with these studies, we found that higher first-trimester FPG was strongly associated with the development of GDM. Higher fasting glycemia level was also association with LGA and assisted vaginal delivery and/or cesarean section.

Our study adds to the literature by showing that FPG at first-trimester could be used as a valuable tool for predicting GDM in a large Chinese population. In addition, we have explored the associations between FPG and adverse pregnancy outcomes.

### Limitations of the study

Firstly, it was a retrospective design and unavoidable selection bias. Secondly, it was a single-center and this may restrict the worldwide application. Third, as the sample size was not large enough, we just used the lower and upper quartiles but not stratified analysis to evaluate the associations between first-trimester FPG and adverse pregnancy outcomes. Thus, further evaluation and studies are still necessary.

## CONCLUSION

Based on our study, we recommend that first-trimester FPG could be used to predict GDM by using the IADPSG criteria. Higher first-trimester FPG was associated with adverse pregnancy outcomes. However, further studies are needed to evaluate the value of first-trimester FPG as a predictor for GDM at multicenter and the usefulness of timely interventions on pregnancy outcome.

### Authors’ Contributions

***Ping Li:*** Data collection, Data analysis and Manuscript writing.

***Shuo Lin:*** Data analysis and manuscript revising.

***Ling Li, Jinhui Cui and Shuisheng Zhou:*** Data collection.

***Jianhui Fan:*** Designed the study and revised the manuscript.
